# Prodromal phase and first episode psychosis in schizophrenia: early signs and diagnosis

**DOI:** 10.1192/j.eurpsy.2022.2008

**Published:** 2022-09-01

**Authors:** A.-G. Buciuc, C. Tapoi, M.-I.-T. Sindrilaru, A. Grecu, C. Petcu

**Affiliations:** 1 “Prof. Dr. Al. Obregia” Clinical Hospital of Psychiatry, General Psychiatry, Bucharest, Romania; 2 Carol Davila University of Medicine and Pharmacy, Psychiatry, Bucharest, Romania

**Keywords:** schizophrénia, prodromal phase, first psychotic episode, Early detection

## Abstract

**Introduction:**

The detection of the initial prodrome of schizophrenia (SK) before the first episode psychosis remains a major concern in current psychiatric research.

**Objectives:**

In this paper we aimed to analyse clinical and psychopathological aspects of prodromes leading to first-lifetime psychotic episodes and to highlight the high-risk features in order to establish preventive strategies and to provide early intervention in SK.

**Methods:**

This is a retrospective observational descriptive study conducted in the ‘Prof. Dr. Alexandru Obregia’ Clinical Hospital of Psychiatry in Bucharest, Romania. We collected data from the medical records of 139 patients previously diagnosed with SK who were admitted to our clinic. For all included patients, diagnoses were recorded according to the DSM-IV TR and ICD-10 classification criteria. Data collected included patient demographics and clinical information. All analyses were conducted using SPSS package.

**Results:**

Of the 139 patients included in this study, 130 patients (93,5%) presented prodromal symptoms. 73 of these patients (52,5%) presented with negative symptoms that were more common in our study in single male patients that had low academic performance and a family history of mental illness, findings consistent with the literature. A decline in social functioning decline was observed in 64 patients (46%) prior to their first admission. 87 patients (62,6%) had a prodromal phase which lasted more than one year.

**Conclusions:**

These findings support the value of early psychopathology in predicting the diagnosis of SK, but clinical guidelines are needed for a more systematic evaluation of the SK prodrome.

**Disclosure:**

No significant relationships.

